# 3-*O*-Methyl-1-isomangostin

**DOI:** 10.1107/S1600536812023409

**Published:** 2012-05-31

**Authors:** Nawong Boonnak, Suchada Chantrapromma, Hoong-Kun Fun

**Affiliations:** a Crystal Materials Research Unit, Department of Chemistry, Faculty of Science, Prince of Songkla University, Hat-Yai, Songkhla 90112, Thailand; b X-ray Crystallography Unit, School of Physics, Universiti Sains Malaysia, 11800 USM, Penang, Malaysia

## Abstract

In the title xanthone derivative [systematic name: 9-hy­droxy-5,10-dimeth­oxy-2,2-dimethyl-11-(3-methyl­but-2-en-1-yl)-2,3,4,12-tetra­hydro-1,7-dioxatetra­phen-12-one], C_25_H_28_O_6_, the xanthone ring system is roughly planar, with an r.m.s. deviation of 0.1038 (1) Å. The chromane ring is in a half-chair conformation and the 3-methyl­but-2-enyl substituent is axially attached with an (+)-anti­clinal conformation. Two weak intra­molecular C—H⋯O inter­actions generate two *S*(6) ring motifs. In the crystal, mol­ecules are linked into ribbons along the *c* axis by O—H⋯O and weak C—H⋯O hydrogen bonds. A π–π inter­action, with a centroid–centroid distance of 3.5413 (8) Å, is also observed.

## Related literature

For background to xanthones and their biological activity, see: Bennett & Lee (1989 ▶); Boonnak *et al.* (2010 ▶); Gopalakrishnan *et al.* (1997 ▶); Ho *et al.* (2002 ▶); Mahabusarakam *et al.* (1987 ▶); Obolskiy *et al.* (2009 ▶); Phongpaichit *et al.* (1994 ▶); Shankaranarayan *et al.* (1979 ▶); Yoshikawa *et al.* (1994 ▶). For related structures, see: Chantrapromma *et al.* (2005 ▶). For details of hydrogen-bond motifs, see: Bernstein *et al.* (1995 ▶). For ring conformations, see: Cremer & Pople (1975 ▶). For bond-length data, see: Allen *et al.* (1987 ▶). For the stability of the temperature controller used in the data collection, see Cosier & Glazer (1986 ▶).
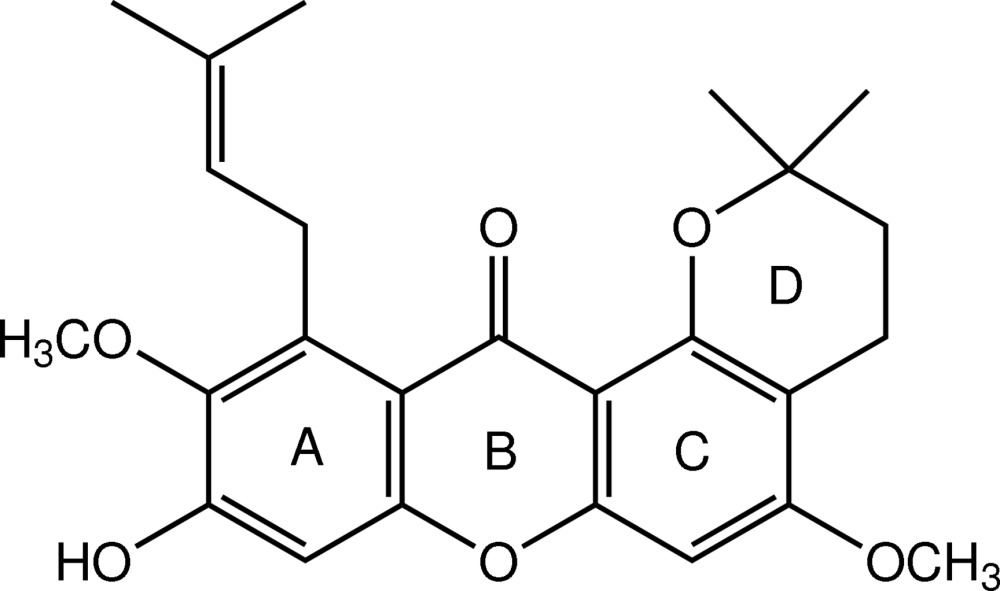


## Experimental

### 

#### Crystal data

C_25_H_28_O_6_
*M* *_r_* = 424.47
Monoclinic, 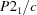

*a* = 10.8635 (9) Å
*b* = 16.6117 (13) Å
*c* = 13.4146 (8) Å
β = 118.843 (5)°
*V* = 2120.5 (3) Å^3^
*Z* = 4
Mo *K*α radiation
μ = 0.09 mm^−1^
*T* = 100 K
0.33 × 0.23 × 0.17 mm

#### Data collection

Bruker APEX DUO CCD area-detector diffractometer
Absorption correction: multi-scan (*SADABS*; Bruker, 2009 ▶) *T*_min_ = 0.970, *T*_max_ = 0.984
21681 measured reflections
5630 independent reflections
4344 reflections with *I* > 2σ(*I*)
*R*_int_ = 0.031

#### Refinement

*R*[*F* ^2^ > 2σ(*F* ^2^)] = 0.046
*wR*(*F* ^2^) = 0.134
*S* = 1.05
5630 reflections
286 parameters
H-atom parameters constrained
Δρ_max_ = 0.42 e Å^−3^
Δρ_min_ = −0.22 e Å^−3^

### 

Data collection: *APEX2* (Bruker, 2009 ▶); cell refinement: *SAINT* (Bruker, 2009 ▶); data reduction: *SAINT*; program(s) used to solve structure: *SHELXTL* (Sheldrick, 2008 ▶); program(s) used to refine structure: *SHELXTL*; molecular graphics: *SHELXTL*; software used to prepare material for publication: *SHELXTL* and *PLATON* (Spek, 2009 ▶).

## Supplementary Material

Crystal structure: contains datablock(s) global, I. DOI: 10.1107/S1600536812023409/rz2760sup1.cif

Structure factors: contains datablock(s) I. DOI: 10.1107/S1600536812023409/rz2760Isup2.hkl

Supplementary material file. DOI: 10.1107/S1600536812023409/rz2760Isup3.cml

Additional supplementary materials:  crystallographic information; 3D view; checkCIF report

## Figures and Tables

**Table 1:** Hydrogen-bond geometry (Å, °)

*D*—H⋯*A*	*D*—H	H⋯*A*	*D*⋯*A*	*D*—H⋯*A*
O5—H1*O*5⋯O2^i^	0.90	1.77	2.6082 (17)	155
C15—H15*A*⋯O1^ii^	0.99	2.55	3.3820 (18)	141
C20—H20*C*⋯O5	0.98	2.57	3.104 (2)	115
C21—H21*A*⋯O2	0.99	2.29	2.807 (2)	111

Symmetry codes: (i) 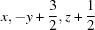; (ii) 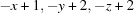.

**Fig. 1.**
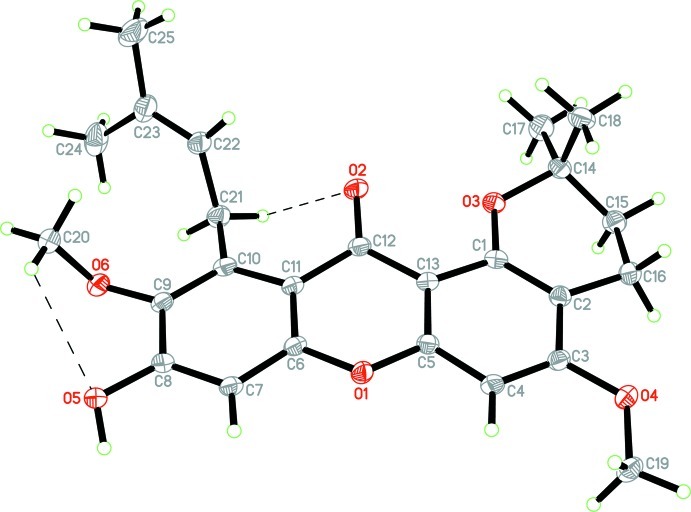
The molecular structure of the title compound, showing 40% probability displacement ellipsoids. Hydrogen bonds were drawn as dashed lines.

**Fig. 2.**
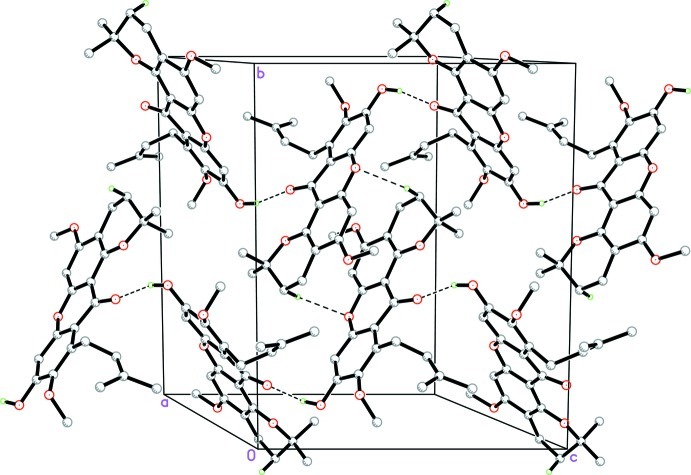
The crystal packing of the title compound viewed approximately along the a axis. Only H atoms involved in hydrogen bonds (dashed lines) are shown for clarity.

## supplementary crystallographic information

### Comment

*Garcinia* genus plants are commonly known as a good providing source of bioactive xanthones (Bennett & Lee, 1989). The *α*-, *β*- and *γ*-mangostins are well known bioactive xanthones that were isolated from *G. mangostana* as major constituents (Mahabusarakam *et al.*, 1987) and they exhibit various biological and pharmacological properties (Obolskiy *et al.*, 2009; Phongpaichit *et al.*, 1994) such as antibacterial (Boonnak *et al.*, 2010), antifungal (Gopalakrishnan *et al.*, 1997), anti-inflammatory (Shankaranarayan *et al.*, 1979), antioxidant (Yoshikawa *et al.*, 1994) and anti-cancer (Ho *et al.*, 2002) activities. These interesting biological properties of xanthones have led us to synthesize the title compound (I) by modification of an isoprenyl side chain of β-mangostin to the chromane ring via acid catalysis (Gopalakrishnan *et al.*, 1997) with the hope to enhance its bioactivity. However our antibacterial testing has found that (I) is less active than its precusor (*β*-mangostin). Herein the crystal structure of (I) is reported.

Compound (I) has a xanthone nucleus with a fused angular fashion chromane ring (Fig. 1). The three-ring system [C1–C13/O1] of the xanthone nucleus is roughly planar with an r.m.s. deviation of 0.1038 (1) Å from the plane through all the fourteen non-hydrogen atoms (maximum deviation of -0.192 (1) Å for atom O19). The chromane ring (C1–C2/C14–C16/O3) is in a half-chair conformation with puckering parameter *Q* = 0.4631 (17) Å, θ = 49.7 (2)° and φ = 272.7 (2)° (Cremer & Pople, 1975), with the puckered C14 and C15 atoms having deviations of -0.306 (1) and 0.293 (2) Å, respectively. The hydroxyl group is planarly attached at atom C8. The two methoxy groups have different orientations in which one methoxy group at atom C3 lies close to the plane of its bound benzene ring with the torsion angle C19–O4–C3–C4 = 1.79 (19)° whereas the other is axially attached at atom C9 with the torsion angle C20–O6–C9–C8 = 80.63 (15)°, indicating an (+)-anti-clinal conformation. The 3-methyl-2-butenyl substituent is attached at atom C10 with the torsion angle C9–C10–C21–C22 = 87.47 (16) °, indicating an (+)-anti-clinal conformation (Fig. 1). Intramolecular C20—H20C···O5 and C21—H21A···O2 weak interactions (Table 1) generate two S(6) ring motifs (Bernstein *et al.*, 1995). The bond distances in (I) are within normal ranges (Allen *et al.*, 1987) and comparable to those found in a related structure (Chantrapromma *et al.*, 2005).

In the crystal packing, the molecules are linked into ribbons along [0 0 1] by O—H···O hydrogen bonds and the adjacent ribbons are further linked by weak C—H···O interactions (Fig. 2 and Table 1). A π–π interaction with the distance of Cg_2_···Cg_2_^ii^ = 3.5413 (8) Å is observed; Cg_2_ is the centroid of C1–C5/C13 ring; symmetry code: (ii) 1-x, 2-y, 2-z.

### Experimental

A solution of *β*-mangostin (30 mg, 0.707 mmol) and *p*-toluenesulfonic acid (75 mg, 0.436 mmol) in dry acetic acid (1.30 ml) was stirred at room temperature for 24 h. The mixture was extracted with ethylacetate. The combined ethylacetate extract was further washed with a saturated aqueous NaHCO_3_ solution and dried over anhydrous MgSO_4_. The solvent was evaporated under reduced pressure to give a yellow residue, which was further purified by column chromatography (hexane/ethylacetate, 9:1 *v*/*v*) to yield the title compound (I). Yellow block-shaped single crystals of the title compound suitable for *X*-ray structure determination were recrystallized from a solution of CHCl_3_/CH_3_OH (9:1 *v*)/*v* by slow evaporation of the solvent at room temperature after several days.

### Refinement

H atoms were positioned geometrically and allowed to ride on their parent atoms, with d(O-H) = 0.90 Å, d(C-H) = 0.95 Å for aromatic and CH, 0.99 for CH_2_ and 0.98 Å for CH_3_ atoms. The *U*_iso_ values were constrained to be 1.5*U*_eq_ of the carrier atom for methyl H atoms and 1.2*U*_eq_ for the remaining H atoms. A rotating group model was used for the methyl groups. One outlier (1 0 0 ) was omitted in the final refinement.

### Crystal data

**Table:**

C_25_H_28_O_6_	*F*(000) = 904
*M**_r_* = 424.47	*D*_x_ = 1.330 Mg m^−^^3^
Monoclinic, *P*2_1_/*c*	Mo *K*α radiation, λ = 0.71073 Å
Hall symbol: -P 2ybc	Cell parameters from 5630 reflections
*a* = 10.8635 (9) Å	θ = 2.1–29.0°
*b* = 16.6117 (13) Å	µ = 0.09 mm^−^^1^
*c* = 13.4146 (8) Å	*T* = 100 K
β = 118.843 (5)°	Block, yellow
*V* = 2120.5 (3) Å^3^	0.33 × 0.23 × 0.17 mm
*Z* = 4	

### Data collection

**Table:**

Bruker APEX DUO CCD area-detector diffractometer	5630 independent reflections
Radiation source: sealed tube	4344 reflections with *I* > 2σ(*I*)
Graphite monochromator	*R*_int_ = 0.031
φ and ω scans	θ_max_ = 29.0°, θ_min_ = 2.1°
Absorption correction: multi-scan (*SADABS*; Bruker, 2009)	*h* = −14→14
*T*_min_ = 0.970, *T*_max_ = 0.984	*k* = −22→16
21681 measured reflections	*l* = −18→18

### Refinement

**Table:**

Refinement on *F*^2^	Primary atom site location: structure-invariant direct methods
Least-squares matrix: full	Secondary atom site location: difference Fourier map
*R*[*F*^2^ > 2σ(*F*^2^)] = 0.046	Hydrogen site location: inferred from neighbouring sites
*wR*(*F*^2^) = 0.134	H-atom parameters constrained
*S* = 1.05	*w* = 1/[σ^2^(*F*_o_^2^) + (0.0637*P*)^2^ + 0.7665*P*] where *P* = (*F*_o_^2^ + 2*F*_c_^2^)/3
5630 reflections	(Δ/σ)_max_ = 0.001
286 parameters	Δρ_max_ = 0.42 e Å^−^^3^
0 restraints	Δρ_min_ = −0.22 e Å^−^^3^

### Special details

**Table:**

Experimental. The crystal was placed in the cold stream of an Oxford Cryosystems Cobra open-flow nitrogen cryostat (Cosier & Glazer, 1986) operating at 100.0 (1) K.
Geometry. All esds (except the esd in the dihedral angle between two l.s. planes) are estimated using the full covariance matrix. The cell esds are taken into account individually in the estimation of esds in distances, angles and torsion angles; correlations between esds in cell parameters are only used when they are defined by crystal symmetry. An approximate (isotropic) treatment of cell esds is used for estimating esds involving l.s. planes.
Refinement. Refinement of F^2^ against ALL reflections. The weighted R-factor wR and goodness of fit S are based on F^2^, conventional R-factors R are based on F, with F set to zero for negative F^2^. The threshold expression of F^2^ > 2sigma(F^2^) is used only for calculating R-factors(gt) etc. and is not relevant to the choice of reflections for refinement. R-factors based on F^2^ are statistically about twice as large as those based on F, and R- factors based on ALL data will be even larger.

### Fractional atomic coordinates and isotropic or equivalent isotropic displacement parameters (Å^2^)

**Table:**

	*x*	*y*	*z*	*U*_iso_*/*U*_eq_	
O1	0.47611 (10)	0.79136 (6)	1.00159 (8)	0.0224 (2)	
O2	0.72643 (12)	0.86788 (7)	0.87266 (9)	0.0303 (2)	
O3	0.56536 (10)	0.99916 (6)	0.79679 (8)	0.0246 (2)	
O4	0.17200 (10)	1.01564 (6)	0.85893 (9)	0.0254 (2)	
O5	0.79450 (11)	0.58712 (6)	1.21935 (9)	0.0269 (2)	
H1O5	0.7523	0.5913	1.2623	0.040*	
O6	0.99052 (11)	0.64398 (6)	1.16684 (9)	0.0271 (2)	
C1	0.48227 (14)	0.96827 (8)	0.83776 (11)	0.0208 (3)	
C2	0.36202 (14)	1.00707 (8)	0.82405 (11)	0.0215 (3)	
C3	0.28457 (14)	0.97195 (8)	0.87218 (11)	0.0214 (3)	
C4	0.32266 (14)	0.89900 (8)	0.92935 (11)	0.0218 (3)	
H4A	0.2671	0.8747	0.9582	0.026*	
C5	0.44460 (14)	0.86281 (8)	0.94284 (11)	0.0199 (3)	
C6	0.60528 (14)	0.75690 (8)	1.03800 (11)	0.0202 (3)	
C7	0.63295 (15)	0.69158 (8)	1.11039 (12)	0.0219 (3)	
H7A	0.5658	0.6742	1.1319	0.026*	
C8	0.76041 (15)	0.65239 (8)	1.15050 (11)	0.0224 (3)	
C9	0.86042 (14)	0.68063 (8)	1.11974 (12)	0.0228 (3)	
C10	0.83498 (15)	0.74805 (8)	1.05092 (12)	0.0227 (3)	
C11	0.70074 (14)	0.78588 (8)	1.00440 (11)	0.0209 (3)	
C12	0.65700 (15)	0.85193 (8)	0.92089 (11)	0.0215 (3)	
C13	0.52913 (14)	0.89451 (8)	0.90017 (11)	0.0199 (3)	
C14	0.50491 (16)	1.06108 (9)	0.70826 (12)	0.0262 (3)	
C15	0.42974 (16)	1.12362 (9)	0.74250 (13)	0.0275 (3)	
H15A	0.4988	1.1504	0.8135	0.033*	
H15B	0.3881	1.1651	0.6823	0.033*	
C16	0.31443 (15)	1.08605 (8)	0.76085 (12)	0.0249 (3)	
H16A	0.2884	1.1237	0.8048	0.030*	
H16B	0.2301	1.0768	0.6862	0.030*	
C17	0.63073 (18)	1.09726 (10)	0.70456 (15)	0.0350 (4)	
H17A	0.6948	1.1207	0.7789	0.052*	
H17B	0.5989	1.1393	0.6461	0.052*	
H17C	0.6798	1.0551	0.6863	0.052*	
C18	0.40695 (18)	1.01862 (10)	0.59661 (13)	0.0319 (3)	
H18A	0.4595	0.9774	0.5806	0.048*	
H18B	0.3689	1.0580	0.5346	0.048*	
H18C	0.3296	0.9933	0.6030	0.048*	
C19	0.09221 (16)	0.98457 (9)	0.91024 (14)	0.0287 (3)	
H19A	0.0136	1.0209	0.8939	0.043*	
H19B	0.1529	0.9805	0.9928	0.043*	
H19C	0.0557	0.9311	0.8790	0.043*	
C20	0.98891 (17)	0.57034 (9)	1.11071 (13)	0.0294 (3)	
H20A	1.0844	0.5485	1.1443	0.044*	
H20B	0.9538	0.5807	1.0295	0.044*	
H20C	0.9274	0.5314	1.1199	0.044*	
C21	0.95680 (15)	0.78156 (9)	1.03784 (13)	0.0269 (3)	
H21A	0.9428	0.8402	1.0236	0.032*	
H21B	1.0449	0.7738	1.1104	0.032*	
C22	0.97349 (17)	0.74360 (9)	0.94364 (14)	0.0291 (3)	
H22A	0.8942	0.7445	0.8697	0.035*	
C23	1.09098 (19)	0.70835 (9)	0.95472 (17)	0.0364 (4)	
C24	1.22440 (18)	0.69855 (11)	1.06549 (19)	0.0455 (5)	
H24A	1.2082	0.7148	1.1284	0.068*	
H24B	1.2981	0.7324	1.0651	0.068*	
H24C	1.2540	0.6421	1.0752	0.068*	
C25	1.0935 (2)	0.67564 (13)	0.8511 (2)	0.0533 (5)	
H25A	1.0008	0.6827	0.7843	0.080*	
H25B	1.1172	0.6183	0.8620	0.080*	
H25C	1.1641	0.7047	0.8395	0.080*	

### Atomic displacement parameters (Å^2^)

**Table:**

	*U*^11^	*U*^22^	*U*^33^	*U*^12^	*U*^13^	*U*^23^
O1	0.0212 (5)	0.0229 (5)	0.0282 (5)	0.0027 (4)	0.0158 (4)	0.0046 (4)
O2	0.0344 (6)	0.0341 (6)	0.0357 (6)	0.0082 (4)	0.0275 (5)	0.0088 (4)
O3	0.0271 (5)	0.0253 (5)	0.0274 (5)	0.0018 (4)	0.0179 (4)	0.0058 (4)
O4	0.0235 (5)	0.0275 (5)	0.0287 (5)	0.0051 (4)	0.0154 (4)	0.0024 (4)
O5	0.0339 (6)	0.0277 (5)	0.0280 (5)	0.0090 (4)	0.0219 (5)	0.0073 (4)
O6	0.0252 (5)	0.0314 (5)	0.0277 (5)	0.0079 (4)	0.0152 (4)	0.0027 (4)
C1	0.0233 (7)	0.0225 (6)	0.0189 (6)	−0.0020 (5)	0.0120 (5)	−0.0008 (5)
C2	0.0233 (7)	0.0220 (6)	0.0194 (6)	0.0001 (5)	0.0105 (5)	−0.0007 (5)
C3	0.0198 (6)	0.0252 (6)	0.0194 (6)	0.0011 (5)	0.0098 (5)	−0.0024 (5)
C4	0.0209 (7)	0.0257 (6)	0.0230 (6)	−0.0002 (5)	0.0138 (6)	0.0011 (5)
C5	0.0220 (6)	0.0203 (6)	0.0192 (6)	0.0000 (5)	0.0114 (5)	0.0005 (5)
C6	0.0197 (6)	0.0230 (6)	0.0208 (6)	0.0010 (5)	0.0121 (5)	−0.0016 (5)
C7	0.0248 (7)	0.0236 (6)	0.0235 (6)	0.0012 (5)	0.0165 (6)	0.0003 (5)
C8	0.0273 (7)	0.0234 (6)	0.0198 (6)	0.0034 (5)	0.0139 (6)	0.0003 (5)
C9	0.0220 (7)	0.0270 (7)	0.0224 (6)	0.0040 (5)	0.0132 (6)	−0.0007 (5)
C10	0.0243 (7)	0.0256 (6)	0.0232 (6)	0.0008 (5)	0.0156 (6)	−0.0016 (5)
C11	0.0225 (7)	0.0230 (6)	0.0218 (6)	0.0016 (5)	0.0143 (5)	−0.0002 (5)
C12	0.0241 (7)	0.0230 (6)	0.0217 (6)	0.0002 (5)	0.0144 (6)	−0.0010 (5)
C13	0.0215 (6)	0.0220 (6)	0.0189 (6)	−0.0003 (5)	0.0119 (5)	−0.0006 (5)
C14	0.0329 (8)	0.0239 (6)	0.0272 (7)	0.0023 (6)	0.0189 (6)	0.0062 (5)
C15	0.0338 (8)	0.0227 (6)	0.0309 (7)	0.0005 (6)	0.0193 (7)	0.0027 (5)
C16	0.0275 (7)	0.0225 (6)	0.0263 (7)	0.0023 (5)	0.0143 (6)	0.0021 (5)
C17	0.0399 (9)	0.0293 (8)	0.0465 (9)	0.0022 (6)	0.0294 (8)	0.0102 (7)
C18	0.0431 (9)	0.0314 (7)	0.0257 (7)	0.0027 (7)	0.0203 (7)	0.0034 (6)
C19	0.0239 (7)	0.0328 (7)	0.0347 (8)	0.0028 (6)	0.0184 (7)	−0.0002 (6)
C20	0.0323 (8)	0.0290 (7)	0.0301 (7)	0.0092 (6)	0.0176 (7)	0.0043 (6)
C21	0.0256 (7)	0.0284 (7)	0.0322 (7)	0.0002 (6)	0.0182 (6)	0.0013 (6)
C22	0.0312 (8)	0.0305 (7)	0.0353 (8)	0.0045 (6)	0.0237 (7)	0.0056 (6)
C23	0.0428 (9)	0.0264 (7)	0.0596 (11)	0.0074 (6)	0.0403 (9)	0.0122 (7)
C24	0.0344 (9)	0.0391 (9)	0.0749 (14)	0.0098 (7)	0.0358 (10)	0.0170 (9)
C25	0.0694 (14)	0.0468 (11)	0.0768 (14)	0.0169 (10)	0.0616 (13)	0.0112 (10)

### Geometric parameters (Å, º)

**Table:**

O1—C6	1.3689 (16)	C14—C18	1.528 (2)
O1—C5	1.3737 (15)	C15—C16	1.522 (2)
O2—C12	1.2363 (16)	C15—H15A	0.9900
O3—C1	1.3635 (16)	C15—H15B	0.9900
O3—C14	1.4653 (16)	C16—H16A	0.9900
O4—C3	1.3579 (16)	C16—H16B	0.9900
O4—C19	1.4377 (18)	C17—H17A	0.9800
O5—C8	1.3549 (16)	C17—H17B	0.9800
O5—H1O5	0.8949	C17—H17C	0.9800
O6—C9	1.3805 (17)	C18—H18A	0.9800
O6—C20	1.4322 (18)	C18—H18B	0.9800
C1—C2	1.3871 (19)	C18—H18C	0.9800
C1—C13	1.4318 (18)	C19—H19A	0.9800
C2—C3	1.411 (2)	C19—H19B	0.9800
C2—C16	1.5114 (19)	C19—H19C	0.9800
C3—C4	1.3863 (19)	C20—H20A	0.9800
C4—C5	1.3848 (18)	C20—H20B	0.9800
C4—H4A	0.9500	C20—H20C	0.9800
C5—C13	1.3982 (18)	C21—C22	1.499 (2)
C6—C7	1.3890 (19)	C21—H21A	0.9900
C6—C11	1.4005 (19)	C21—H21B	0.9900
C7—C8	1.3826 (19)	C22—C23	1.346 (2)
C7—H7A	0.9500	C22—H22A	0.9500
C8—C9	1.415 (2)	C23—C24	1.502 (3)
C9—C10	1.3917 (19)	C23—C25	1.505 (3)
C10—C11	1.4258 (19)	C24—H24A	0.9800
C10—C21	1.522 (2)	C24—H24B	0.9800
C11—C12	1.4734 (19)	C24—H24C	0.9800
C12—C13	1.4611 (19)	C25—H25A	0.9800
C14—C17	1.516 (2)	C25—H25B	0.9800
C14—C15	1.523 (2)	C25—H25C	0.9800
			
C6—O1—C5	119.66 (11)	H15A—C15—H15B	107.9
C1—O3—C14	117.77 (11)	C2—C16—C15	111.17 (12)
C3—O4—C19	117.28 (11)	C2—C16—H16A	109.4
C8—O5—H1O5	108.7	C15—C16—H16A	109.4
C9—O6—C20	112.70 (11)	C2—C16—H16B	109.4
O3—C1—C2	122.40 (12)	C15—C16—H16B	109.4
O3—C1—C13	116.11 (12)	H16A—C16—H16B	108.0
C2—C1—C13	121.44 (12)	C14—C17—H17A	109.5
C1—C2—C3	118.60 (12)	C14—C17—H17B	109.5
C1—C2—C16	121.55 (13)	H17A—C17—H17B	109.5
C3—C2—C16	119.85 (12)	C14—C17—H17C	109.5
O4—C3—C4	123.32 (12)	H17A—C17—H17C	109.5
O4—C3—C2	114.67 (12)	H17B—C17—H17C	109.5
C4—C3—C2	122.01 (12)	C14—C18—H18A	109.5
C5—C4—C3	117.60 (12)	C14—C18—H18B	109.5
C5—C4—H4A	121.2	H18A—C18—H18B	109.5
C3—C4—H4A	121.2	C14—C18—H18C	109.5
O1—C5—C4	114.08 (12)	H18A—C18—H18C	109.5
O1—C5—C13	121.95 (12)	H18B—C18—H18C	109.5
C4—C5—C13	123.97 (12)	O4—C19—H19A	109.5
O1—C6—C7	114.52 (12)	O4—C19—H19B	109.5
O1—C6—C11	122.16 (12)	H19A—C19—H19B	109.5
C7—C6—C11	123.33 (12)	O4—C19—H19C	109.5
C8—C7—C6	118.57 (13)	H19A—C19—H19C	109.5
C8—C7—H7A	120.7	H19B—C19—H19C	109.5
C6—C7—H7A	120.7	O6—C20—H20A	109.5
O5—C8—C7	122.36 (13)	O6—C20—H20B	109.5
O5—C8—C9	117.91 (12)	H20A—C20—H20B	109.5
C7—C8—C9	119.73 (12)	O6—C20—H20C	109.5
O6—C9—C10	119.36 (13)	H20A—C20—H20C	109.5
O6—C9—C8	118.73 (12)	H20B—C20—H20C	109.5
C10—C9—C8	121.70 (12)	C22—C21—C10	114.13 (13)
C9—C10—C11	118.61 (13)	C22—C21—H21A	108.7
C9—C10—C21	117.73 (12)	C10—C21—H21A	108.7
C11—C10—C21	123.44 (12)	C22—C21—H21B	108.7
C6—C11—C10	117.87 (12)	C10—C21—H21B	108.7
C6—C11—C12	118.90 (12)	H21A—C21—H21B	107.6
C10—C11—C12	123.20 (12)	C23—C22—C21	125.71 (16)
O2—C12—C13	124.11 (12)	C23—C22—H22A	117.1
O2—C12—C11	120.20 (12)	C21—C22—H22A	117.1
C13—C12—C11	115.69 (12)	C22—C23—C24	124.58 (17)
C5—C13—C1	116.31 (12)	C22—C23—C25	119.59 (18)
C5—C13—C12	119.45 (12)	C24—C23—C25	115.83 (15)
C1—C13—C12	124.24 (12)	C23—C24—H24A	109.5
O3—C14—C17	104.25 (12)	C23—C24—H24B	109.5
O3—C14—C15	109.29 (11)	H24A—C24—H24B	109.5
C17—C14—C15	111.26 (12)	C23—C24—H24C	109.5
O3—C14—C18	107.36 (11)	H24A—C24—H24C	109.5
C17—C14—C18	111.32 (13)	H24B—C24—H24C	109.5
C15—C14—C18	112.91 (13)	C23—C25—H25A	109.5
C16—C15—C14	111.83 (12)	C23—C25—H25B	109.5
C16—C15—H15A	109.3	H25A—C25—H25B	109.5
C14—C15—H15A	109.3	C23—C25—H25C	109.5
C16—C15—H15B	109.3	H25A—C25—H25C	109.5
C14—C15—H15B	109.3	H25B—C25—H25C	109.5
			
C14—O3—C1—C2	−18.47 (18)	O1—C6—C11—C12	−4.4 (2)
C14—O3—C1—C13	163.92 (11)	C7—C6—C11—C12	175.70 (12)
O3—C1—C2—C3	−178.12 (12)	C9—C10—C11—C6	5.0 (2)
C13—C1—C2—C3	−0.63 (19)	C21—C10—C11—C6	−169.51 (13)
O3—C1—C2—C16	0.9 (2)	C9—C10—C11—C12	−173.19 (13)
C13—C1—C2—C16	178.37 (12)	C21—C10—C11—C12	12.3 (2)
C19—O4—C3—C4	1.79 (19)	C6—C11—C12—O2	−165.46 (13)
C19—O4—C3—C2	−177.51 (12)	C10—C11—C12—O2	12.8 (2)
C1—C2—C3—O4	177.28 (11)	C6—C11—C12—C13	13.96 (18)
C16—C2—C3—O4	−1.74 (18)	C10—C11—C12—C13	−167.83 (12)
C1—C2—C3—C4	−2.0 (2)	O1—C5—C13—C1	178.29 (12)
C16—C2—C3—C4	178.95 (12)	C4—C5—C13—C1	−1.0 (2)
O4—C3—C4—C5	−176.20 (12)	O1—C5—C13—C12	−1.69 (19)
C2—C3—C4—C5	3.0 (2)	C4—C5—C13—C12	179.04 (13)
C6—O1—C5—C4	−168.53 (12)	O3—C1—C13—C5	179.69 (11)
C6—O1—C5—C13	12.13 (19)	C2—C1—C13—C5	2.05 (19)
C3—C4—C5—O1	179.18 (12)	O3—C1—C13—C12	−0.32 (19)
C3—C4—C5—C13	−1.5 (2)	C2—C1—C13—C12	−177.96 (13)
C5—O1—C6—C7	170.98 (11)	O2—C12—C13—C5	168.33 (13)
C5—O1—C6—C11	−8.95 (19)	C11—C12—C13—C5	−11.06 (18)
O1—C6—C7—C8	179.30 (12)	O2—C12—C13—C1	−11.6 (2)
C11—C6—C7—C8	−0.8 (2)	C11—C12—C13—C1	168.96 (12)
C6—C7—C8—O5	−178.50 (12)	C1—O3—C14—C17	165.58 (12)
C6—C7—C8—C9	1.6 (2)	C1—O3—C14—C15	46.56 (16)
C20—O6—C9—C10	−104.50 (15)	C1—O3—C14—C18	−76.23 (15)
C20—O6—C9—C8	80.63 (15)	O3—C14—C15—C16	−58.35 (16)
O5—C8—C9—O6	−4.17 (19)	C17—C14—C15—C16	−172.94 (12)
C7—C8—C9—O6	175.69 (12)	C18—C14—C15—C16	61.06 (16)
O5—C8—C9—C10	−178.92 (12)	C1—C2—C16—C15	−13.47 (18)
C7—C8—C9—C10	0.9 (2)	C3—C2—C16—C15	165.52 (12)
O6—C9—C10—C11	−179.05 (12)	C14—C15—C16—C2	41.79 (16)
C8—C9—C10—C11	−4.3 (2)	C9—C10—C21—C22	87.47 (16)
O6—C9—C10—C21	−4.18 (19)	C11—C10—C21—C22	−97.93 (16)
C8—C9—C10—C21	170.53 (13)	C10—C21—C22—C23	−124.59 (16)
O1—C6—C11—C10	177.31 (12)	C21—C22—C23—C24	2.4 (3)
C7—C6—C11—C10	−2.6 (2)	C21—C22—C23—C25	−177.60 (16)

### Hydrogen-bond geometry (Å, º)

**Table:**

*D*—H···*A*	*D*—H	H···*A*	*D*···*A*	*D*—H···*A*
O5—H1*O*5···O2^i^	0.90	1.77	2.6082 (17)	155
C15—H15*A*···O1^ii^	0.99	2.55	3.3820 (18)	141
C20—H20*C*···O5	0.98	2.57	3.104 (2)	115
C21—H21*A*···O2	0.99	2.29	2.807 (2)	111

Symmetry codes: (i) *x*, −*y*+3/2, *z*+1/2; (ii) −*x*+1, −*y*+2, −*z*+2.
